# Polymorphisms in the glutathione pathway modulate cystic fibrosis severity: a cross-sectional study

**DOI:** 10.1186/1471-2350-15-27

**Published:** 2014-03-04

**Authors:** Fernando Augusto de Lima Marson, Carmen Silvia Bertuzzo, Antonio Fernando Ribeiro, Jose Dirceu Ribeiro

**Affiliations:** 1 Center for Investigation in Pediatrics, Faculty of Medical Sciences, University of Campinas. Tessália Vieira de Camargo, 126. Cidade Universitária “Zeferino Vaz”, CEP: 13083-887 Campinas, São Paulo, Brazil; 2 Department of Medical Genetics, Faculty of Medical Sciences, University of Campinas. Tessália Vieira de Camargo, 126. Cidade Universitária “Zeferino Vaz”, CEP: 13083-887 Campinas, São Paulo, Brazil

**Keywords:** Cystic fibrosis, CFTR, GSH, GCLC, GST, Genotype, Phenotype, Modifier genes

## Abstract

**Background:**

Cystic fibrosis (CF) clinically manifests with various levels of severity, which are thought to be modulated by mutations in the cystic fibrosis transmembrane conductance regulator gene (*CFTR)*, modifier genes, and the environment. This study verified whether polymorphisms in modifier genes associated with glutathione (GSH) metabolism influence CF severity.

**Methods:**

A cross-sectional study of 180 CF patients was carried out from 2011 to 2012. We analyzed *CFTR* mutations, polymorphisms (*GSTM1* and *GSTT1* deletions, *GSTP1* + 313A > G, *GCLC*-129C > T, and *GCLC*-3506A > G) in modifier genes and CF clinical severity as assessed by 28 clinical and laboratory variables.

**Results:**

Significant associations were found between modifier gene polymorphisms and particular phenotypes or genotype changes. These included GCLC-129C > T with a higher frequency of the *Pseudomonas aeruginosa* mucoid to CC genotype (*p* = 0.044), and *GCLC*-3506A > G with a higher frequency of the no-mucoid *P. aeruginosa* (NMPA) to AA genotype (*p* = 0.012). The *GSTT1* deletion was associated with a higher frequency of the NMPA to homozygous deletion (*p* = 0.008), *GSTP1* + 313A > G with a minor risk of osteoporosis (*p* = 0.036), and patient age ≤ 154 months (*p* = 0.044) with the AA genotype. The Bhalla score was associated with *GCLC*-3506A > G (*p* = 0.044) and *GSTM1*/*GSTT1* deletion polymorphisms (*p* = 0.02), while transcutaneous hemoglobin oxygen saturation levels were associated with *GSTT1* deletions (*p* = 0.048).

**Conclusion:**

CF severity is associated with polymorphisms in GSH pathways and *CFTR* mutations.

## Background

Cystic fibrosis (CF) presents with broad phenotypic variability, even in patients with identical mutations in the causative gene, cystic fibrosis transmembrane conductance regulator (*CFTR*) [1]. Explanations for this include environmental factors [2], medical management [3], nutritional status [4], emotional maladjustments [5], socioeconomic status [3], *CFTR* mutations [1], and modifier genes [1,3,6]. In this context, CF modifier genes have been studied with the aim of increasing chlorine transport and/or controlling pulmonary inflammation and infection [6-9].

Our group studied CF severity in association with several modifier genes including polymorphisms in the genes: *MBL-2, TGF-*β*1*, *CD14*[10], *ACE*[11], *ADRB2*[12], *TCF7L2*[13], *ADRA2A*[14], *COX-2*[15] and *IFRD1*[16]. These polymorphisms were associated with clinical variables including lung and digestive disease.

Glutathione (GSH) is a tripeptide composed of L-cysteine, L-glutamic acid, and glycine. It is a crucial part of the intracellular defense system, which protects the epithelium against the injuries and inflammation [17] common to CF that are caused by oxidation [18]. As polymorphisms can alter the GSH metabolic pathway, genetic variations of this pathway have previously been studied in association with CF [19-21].

The glutathione S-transferase (GST) family of enzymes comprises proteins with distinct genetic origins that form a detoxification system, which protects the human body against electrophilic compounds and oxidative stress [22]. The GST protein is responsible for combining compounds that cause oxidative stress with GSH. It is therefore possible that *GST* polymorphisms are involved in CF severity [18,22], especially with regard to pulmonary disease.

Genetic variants of the *GST* genes include glutathione S-transferase mu 1 (*GSTM1*) located on chromosome 1p13.3, and glutathione S-transferase theta 1 (*GSTT1*) on chromosome 22q11.23 [23], which both exhibit polymorphic deletions [22,24]. The null *GST* allele does not encode a GST protein, so homozygous genotypes are associated with increased CF clinical severity [25,26]. The glutathione S-transferase pi gene *(GSTP1)* on chromosome 11q13 [23] is associated with xenobiotic metabolism and susceptibility to cancer and other diseases [22]. Its most commonly studied polymorphism is an A → G base exchange at the +313 position (substituting isoleucine by valine at codon 105) [27].

The glutamate-cysteine ligase, catalytic subunit gene (*GCLC*) on chromosome 6p12 [23] encodes the catalytic subunit of glutamate-cysteine ligase (GCL), which is the first limiting enzyme in GSH synthesis [28]. The GCL holoenzyme is a heterodimer of approximately 104 kDa composed of catalytic-GCLC and regulatory-GCLR subunits [18]. The -129C > T and -3506A > G polymorphisms of *GCLC* are located in the promoter region and are responsible for reduced production of GSH [18,28].

Of these genes, *GSTP1* is associated with hepatic disease [19] and infection [20], *GSTM1* with greater CF clinical severity [21], *GSTT1* with no CF clinical variables, while *GCLC* has not been previously studied in relation to CF. However, as the action of the GSH protein is closely related to that of CFTR [29], it is conceivable that *GCLC* and *GST* polymorphisms influence CF severity [19-21,26,30]. This study therefore aimed to determine whether genetic polymorphisms in the GSH metabolic pathway are associated with CF severity under different phenotypes of the disease.

## Methods

This cross-sectional study was conducted in a university center for CF care between 2011 and 2012. Two hundred and fifteen patients were selected for the study, of which 35 were excluded for not signing the consent form or because of a lack of clinical data for statistical analysis. CF diagnosis was confirmed if levels of chloride in the sweat exceeded 60 mEq/L and by *CFTR* mutation screening when possible. CF patients, with no identified *CFTR* mutation or with one *CFTR* mutation screened, were classified as CF disease, considering: (i) all patients had levels of chloride in the sweat exceeded 60 mEq/L; (ii) CF clinical symptoms were diagnosed in all patients as: chronic obstructive pulmonary disease, bacteria in sputum, spirometry with obstruction values for forced expiratory volume in the first second (FEV_1_%), associated comorbidities (i.e. osteoporosis, nasal polyps, diabetes mellitus and pancreatic insufficiency); (iii) the dosage of active CFTR in epithelium via rectal biopsy was performed - all patients included had abnormal values for biopsy – absence of active CFTR was found; (iv) nasal potential was realized in some patients - all values were changed – but the comparison was not performed, taking into account a control standard curve, being an inconclusive data. By this method was possible to exclude Cystic Fibrosis Related Diseases.

No patients were diagnosed by a neonatal screening test. Patient DNA was obtained by phenol-chloroform extraction and 50 ng/mL was used for analysis as evaluated by a GE NanoVue™ Spectrophotometer (GE Healthcare Biosciences, Pittsburgh, PA, USA).

### Clinical variables

Several clinical variables were employed, including Shwachman-Kulczycki, Kanga and Bhalla clinical scores [31]; body mass index (BMI) [for patients older than 19 years, the BMI = weight/(height)^2^ formula was used, while remaining patients used the WHO ANTHRO program (children 0–5 years of age) or the WHO ANTHRO PLUS program (children 5–19 years of age)]; patient’s age (≤154 and >154 months); time to diagnosis (≤24 and >24 months); time of first clinical symptoms (digestive: ≤3 and >3 months; pulmonary: ≤6 and >6 months); time to first colonization by *Pseudomonas aeruginosa* (≤31 and >31 months); bacteria in the respiratory airways: mucoid *P. aeruginosa* and no mucoid *P. aeruginosa*, *Achromobacter xylosoxidans, Burkholderia cepacia* and *Staphylococcus aureus -* the positive status was evaluated considering chronic infection (patients in whom more than 50% of the preceding 12 months was culture positive) + intermittent infection (patients with less than 50% of cultures positive). A patient was negative considering as free of bacterium (when no bacterium was grown from samples in the previous 12 months, despite a history of prior colonization) + never infected (patients in whom the bacterium) has never been cultured, i.e. this consensus was formulated for *P. aeruginosa*, but in our data was used for all bacteria [32]; transcutaneous hemoglobin oxygen saturation (SpO2) and spirometry variables.

Spirometry was performed in patients older than seven years of age with the CPFS/D spirometer (MedGraphics, Saint Paul, MN, USA) and data were recorded using the PF BREEZE software version 3.8B for Windows 95/98/NT [33]. The following variables were included: forced vital capacity [FVC(%)]; forced expiratory volume in the first second [FEV_1_(%)]_,_ the ratio between FEV_1_ and FVC(%) [FEV_1_/FVC(%)]; and forced expiratory flow between 25 and 75% of the FVC [FEF_25-75_%]. The data was analyzed considering international curves values for spirometry tests [34,35].

The comorbidities analyzed were nasal polyps, osteoporosis, meconium ileus, diabetes mellitus, and pancreatic insufficiency. This study was approved by the Institutional Ethics Committee from the Faculty of Medical Sciences, University of Campinas (#528/2008), and all included patients or their parents signed a consent form before beginning the study.

### *CFTR* mutation identification

*CFTR* mutation identification was performed by polymerase chain reaction (PCR) for F508del and the fragment-length polymorphism method for G542X, R1162X, R553X, G551D, and N1303K mutations. Some CF mutations were identified by sequencing or Multiplex Ligation-dependent Probe Amplification (MLPA) analysis: S4X, 2183A > G, 1717-G > A, and I618T. A MegaBace1000® sequencer (GE Healthcare Biosciences) was used for sequencing and MLPA.

The *CFTR* genotype was used as a correction factor for statistical analysis. All class I, II or III mutations, but not class IV mutations (P205S and R334W), identified were included in statistical analysis.

### Identification of polymorphisms associated with GSH metabolic pathway genes

Polymorphism identification was carried out using PCR analysis. For *GSTM1* and *GSTT1* genes, a multiplex PCR reaction was performed using the *CYP1A1* gene as an internal amplification control [36]. *GCLC*-129C > T, -3506A > G [18,28] and *GSTP1* + 313A > G [27] polymorphisms were identified by PCR followed by enzymatic digestion.

### Statistical analysis

Statistical analysis was performed using Statistical Package for Social Sciences (SPSS) software version 21.0 (SPSS Inc., Chicago, IL, USA), Epi Info version 6.0 [37] and R version 2.12 (Comprehensive R Archive Network, 2011). GPower 3.0.3.1 software [38] was used to calculate the statistical power, which was required to be above 80% for analysis.

Statistical tests included the analysis of variance (ANOVA) and the chi-square (χ^2^) test (Odds Ratio -OR) for *GSTM1* and *GSTT1*, and the *t*-test and Fisher’s exact test for *GCLC*-129C > T, *GCLC*-3506A > G and *GSTP1* + 313A > G polymorphisms. To avoid spurious data caused by the performance of multiple tests [39], the significance level (α) was adjusted by the Bonferroni correction (α _corrected_ = 0.05/number of tests → 0.05/4 = 0.0125). The value of α was corrected considering clinical marker analysis of the same group of patients, taking into account, the *CFTR* mutation genotype.

Data distribution showing a high standard deviation was analyzed in groups distributed according to median value. Variables that were adjusted by median to short (more severe) and longtime were patient’s age, time to diagnosis, onset of pulmonary and digestive symptoms, and time to the first isolation of *P. aeruginosa.*

Analyses were performed of four cohorts: (i) all patients with CF (n = 180); (ii) patients with no identified *CFTR* mutation (n = 44); (iii) patients with an identified mutant *CFTR* allele (Class I, II and/or III) (n = 51); and (iv) patients with two identified *CFTR* mutations (Class I, II and/or III) (n = 85). For (ii) and (iii) groups, a second analysis was performed. In this case, CF patients with pancreatic sufficiency (PS) were excluded. Patients with mutations Class I, II and III for *CFTR* gene have severe disease, strongly associated with pancreatic insufficiency (PI). Excluding PI patients was a method to associated different *CFTR* mutation groups with no atypical CF – associated less severe mutation (Class IV, V and VI). After exclusion, we have in (ii) and (iii) groups, respectively, 35 and 43 CF patients.

## Results and discussion

One of the most intriguing aspects of CF is that patients with the same *CFTR* genotype can present with phenotypic differences [40]. At our CF center, all patients receive free medication provided by the state, have a similar socioeconomic status, share similar Class I, II and/or III mutations, receive support from the Cystic Fibrosis Association (http://www.fibrocis.org.br/), and there are no severe cases of malnutrition. This therefore makes our sample more phenotypically homogeneous for studies involving gene modulation characteristics.

Variations in CF severity can be associated with a modifier gene, such as those associated with oxidative stress [19-21,26,30] that are related to chronic obstructive pulmonary disease (COPD) [18]. The COPD pathophysiology is similar, in some aspects, to CF in that it involves cellular responses, inflammatory mediators, and oxidative stress [41]. However, there is no mention in the scientific literature of *GCLC* polymorphisms as clinical modulators of CF severity, and is necessary new studies to illuminate about GST genes and CF severity.

One of the main functions of GSH is to detoxify xenobiotics and their metabolites, and this function is dependent on GST proteins. The *GST* gene family has been linked with several diseases [22], as *GSTM1, GSTT1* and *GSTP1* polymorphisms were found to be associated with cancer, drugs, chemotherapy resistance [42], and respiratory diseases such as asthma [30]. For example, expression of the variant form of *GSTP1* (where isoleucine is substituted for valine at codon 105) results in lower enzymatic activity, which is a risk factor for the development of cancer and pulmonary diseases such as CF [43].

The effects of *GSTM1, GSTT1* and *GSTP1* polymorphisms on spirometry were previously investigated in 1,940 children (aged 8–11 years) [44]. The null *GSTM1* genotype was associated with a decrease in annual FVC(%) and FEV_1_(%) gain; likewise, homozygosity for the *GSTP1* allele was linked with slower spirometric gain for the same markers. The *GSTM1* and *GSTP1* genotypes therefore appear to be associated with spirometric evolution, and could increase the severity of diseases of pulmonary obstruction, depending on the genotype and gene combination.

Table 1 shows the *GCLC*, *GSTM1, GSTT1* and *GSTP1* polymorphism distribution according to genotype in the present study. The -129C > T polymorphism in the promoter region of *GCLC* stimulates different responses to oxidative stress by decreasing GSH production and reducing cellular antioxidant capacity [45]. In the present study, it was associated with a higher frequency of the mucoid *P. aeruginosa* to CC genotype for *GCLC*-129C > T polymorphism in patients with one *CFTR* mutation identified (Table 2; *p* = 0.044). This association may be related to the lower GCLC protein expression in CC genotypes, which reduces circulating GSH levels. The T allele is also associated with increased GSH expression, as described in protein expression studies on cardiovascular disease [45]. In the cited literature, we only found one study that associated the *GCLC* polymorphism with CF severity. In this previous study, the GAG micro-satellite *GCLC* polymorphism was analyzed in 440 CF patients, and *CFTR* mutations of lower gravity and highest number of GAG repeats in the *GCLC* gene were associated with higher values of FEV_1_(%) [20].

**Table 1:** **Distribution of *GCLC*, *GSTM1, GSTT1 *and *GSTP1 *polymorphisms**

**Gene**	**Polymorphism**		**Genotypes (N analyzed and %)**		**Grouping (N analyzed and %)**		**Total**
*GCLC*	−129C > T^a^	CC	CT	TT	CC	CT + TT	181 (100%)
		145 (80.11%)	29 (16.02%)	7 (3.87%)	145 (80.11%)	36 (19.89%)	
	−3506A > G^b^	AA	AG	GG	AA	AG + GG	181 (100%)
		119 (65.75%)	56 (30.94%)	6 (3.31%)	119 (65.75%)	62 (35.25%)	
*M1*	Deletion^c^	-	+				181 (100%)
		73 (40.33%)	108 (59.67%)				
*T1*	Deletion^d^	-	+				181 (100%)
		63 (34.81%)	118 (65.19%)				
*M1/T1*	Deletion	−/−	+/− and −/+	+/+			181 (100%)
		19 (10.50%)	99 (54.70%)	63 (34.80%)			
*GSTP1*	+313A > G^f^	AA	AG	GG	AA	AG + GG	181 (100%)
		98 (54.14%)	74 (40.88%)	9 (4.98%)	98 (54.14%)	83 (45.86%)	

*GSTM1*, Glutathione S-transferase Mu; *GSTT1*, Glutathione S-transferase Theta 1; *GSTP1*, Glutathione S-transferase Pi 1; *GCLC*, Glutamate-cysteine ligase, catalytic subunit; N, Sample size; −, Null allele; +, Expressed allele.

The statistical association, taking into account the *CFTR* mutation groups, with the polymorphisms distribution was by p-values in the table: ^a^0.880 (*GCLC*-129C > T); ^b^0.075 *GCLC*-3506A > G); ^c^0.969 (M1); ^d^0.088 (T1); ^e^0,329 (*GSTP1* + 313A > G)^f^.

**Table 2:** Polymorphisms in modifier genes associated with categorical variables of cystic fibrosis severity

***CFTR *** **group**	**Polymorphism**	**Genotype**	**Variable**			**p**^**c**^	**OR**	**CI (5–95%)**
One *CFTR* mutation identified	GCLC-129C > T		PAM					
			Presence	Absence	Total			
		CC	25	17	42	**0.044**	**11.27**	**1.6–272.6**
		CT + TT	1	8	9		-	-
	GCLC-3506A > G		PANM					
			Presence	Absence	Total			
		AA	28	9	37	**0.012**	**7.408**	**1.905–33.43**
		AG + GG	4	10	14		-	-
No mutation identified	*GSTT1* gene deletion		PANM					
			Presence	Absence	Total			
		Not expressed	13	9	21	**0.008**	**7.895**	**2.095–34.96**
		Expressed	4	23	27		-	-
One *CFTR* mutation identified + IP	*GSTM1* gene deletion		Digestive symptoms					
			< 6 months	≥ 6 months				
		Not expressed	3	12	15	**0.032**	**0.134**	**0.023-0.606**
		Expressed	14	7	21		**-**	**-**
Two mutations identified	GSTP1 + 313A > G		Osteoporosis					
			Presence	Absence	Total			
		AA	2	42	44	**0.036**	**0.141**	**0.028–0.687**
		AG + GG	9	26	35		-	-
Without taking *CFTR* mutation into account			Age (months)					
			≤ 154	> 154	Total			
		AA	58	39	97	**0.044**	**2.198**	**1.208–4.037**
		AG + GG	33	49	82		-	-

Statistical analysis was performed by Fisher’s exact test. *CFTR*, Cystic fibrosis transmembrane regulator; *GCLC*, Glutamate-cysteine ligase catalytic subunit; *GSTM1*, Glutathione S-transferase mu 1; *GSTT1*, Glutathione S-transferase theta 1; *GSTP1*, Glutathione S-transferase Pi 1; PI, Pancreatic insufficiency; PAM, *Pseudomonas aeruginosa* mucoid; PANM, *Pseudomonas aeruginosa* no mucoid; p^c^, *P*-value corrected by Bonferroni test; OR, Odds ratio; CI, Confidence interval.

The *GCLC*-3506A > G polymorphism is not in Hardy-Weinberg equilibrium as shown in Table 3, which also shows the complete genotypic characteristics of *GCLC*, *GSTM1*, *GSTT1*, and *GSTP1* polymorphisms and *CFTR* mutations in CF patients with regard to chromosomal position, polymorphism location within the gene, and minor allele frequency. *GCLC*-3506A > G was associated with a higher frequency of the no mucoid *P. aeruginosa* to AA genotype, and with a lower frequency of the no mucoid *P. aeruginosa* to AG + GG genotype group in patients with one *CFTR* mutation identified (Table 2; *p* = 0.012) and higher Bhalla score values (without taking *CFTR* mutation into account; *p* = 0.044)*.*

**Table 3:** **Genotyping of *GCLC*, *GSTM1*, *GSTT1*, and *GSTP1 *polymorphisms and *CFTR *mutations**

**Gene**	**Chromosomal position**	**Location**	**Polymorphism**	**MAF**	**HWE**	***p*-value**^**a**^
*GCLC,* rs17883901	6p12	Promoter region	C > T	0.12	9.97	<0.005
*GCLC*, rs137852340	6p12	Promoter region	A > G	0.19	0.04	>0.05
*GSTP1*, rs1695	11q13	Exon	A > G	0.25	1.11	>0.05
*GSTM1*	1p13.3		Deletion			
*GSTT1*	22q11.23		Deletion			
***CFTR *** **mutation**	**N**	**Frequency**				
F508del/F508del	57	31.67%				
F508del/G542X	12	6.67%				
F508del/R1162X	5	2.78%				
F508del/N1303K	4	2.22%				
F508del/R553X	1	0.56%				
F508del/S4X	1	0.56%				
F508del/1717-1G > A	1	0.56%				
G542X/R1162X	1	0.56%				
G542X/I618T	1	0.56%				
G542X/2183A > G	1	0.56%				
R1162X/R1162X	1	0.56%				
F508del/-	45	25.00%				
G542X/-	5	2.78%				
R1162X/-	1	0.56%				
−/−	44	24.45%				

MAF, Minor allele frequency; HWE, Hardy Weinberg Equilibrium; ^a^*P*-value for Hardy-Weinberg Equilibrium; N, Number of patients; −, No identified *CFTR* mutation.

The Bhalla score is associated with an impairment of the pulmonary parenchyma structure and higher values characterize major changes in thoracic tomography. Unexpectedly, we also found that the greatest expression of the A allele in the *GCLC*-3506A > G polymorphism did not protect against no mucoid *P. aeruginosa* colonization. However, protection against lung deterioration was evident when we considered the Bhalla score. This score was also associated with *GSTM1*/*GSTT1* deletions (*p* = 0.02), with a lower frequency of heterozygous compared with homozygous deletions. Moreover, the *GSTT1* deletion was found to be associated with SpO2 values (*p* = 0.048; Table 4).

**Table 4:** Polymorphisms in modifier genes associated with numerical variables of cystic fibrosis severity

***CFTR *** **group**	**Variable**	**Polymorphism**	**Genotype**	**N**	**Mean**	**SD**	**SEM**	***p*- value corrected**
Without taking *CFTR* mutation into account	Bhalla score^a^	GCLC-3506A > G	AA	94	19.70	6.007	0.620	**0.044**
			AG + GG	43	17.00	5.033	0.768	
No mutation identified	SpO2^a^	*GSTT1* deletion	Not expressed	15	96.13	2.232	0.576	**0.048**
			Expressed	29	93.17	5.245	0.974	
	Bhalla score^b^	*GSTM1*/*GSTT1* deletions	−/−	4	14.75	1.258	0.629	**0.02**
			+/− and −/+	21	6.900	6.610	1.442	
			+/+	9	15.33	7.533	2.511	

^a^Using Student’s *t*-test; ^b^Using analysis of variance.

*CFTR*, Cystic fibrosis transmembrane regulator; SpO2, Hemoglobin oxygen saturation in the blood; −, Null allele; +, Expressed allele; N, Number of patients.

*GCLC* haplotype analysis for *GCLC*-129C > T and *GCLC*-3506A > G showed association for *A. xylosoxidans* and CC + AA genotypes (OR = 17.9; CI95% = 2.781-411.6; Table 5).

**Table 5:** GCLC-129C > T and GCLC-3506A > G haplotype polymorphisms in modifier genes associated with categorical variables of cystic fibrosis severity

***CFTR *** **group**	**PI taking into account**	**Haplotype**	**Genotype**	**Variable**			**p**^**c**^	**OR**	**CI (5–95%)**
				**AX**					
				**Presence**	**Absence**	**Total**			
Two mutation identified	No	GCLC-129C > T + GCLC-3506A > G	CC + AA	10	26	36		**17.9**	**2.781-411.6**
			CC + (AG or GG)	1	30	31	**0.024**	**0.149**	**0.007-0.959**
			(CT or TT) + GG	0	13	13		-	-
			TT + GG	0	5	5		-	-

Statistical analysis was performed by χ^2^ test. *CFTR*, Cystic fibrosis transmembrane regulator; *GCLC*, Glutamate-cysteine ligase catalytic subunit; PI, Pancreatic insufficiency; AX, Achromobacter xylosoxidans; p^c^, *P*-value corrected by Bonferroni test; OR, Odds ratio; CI, Confidence interval.

The present study found that the AA genotype of the *GSTP1* + 313A > G polymorphism was associated with a low risk of osteoporosis (*p* = 0.036; with two *CFTR* mutations identified) as a protective factor and with young age ≤ 154 months (*p* = 0.044; without taking the *CFTR* gene into account) as a risk factor. The G allele, however, is responsible for increased *GSTP1* expression. The presence of osteoporosis is influenced by several different factors, including mutations in the *CFTR* gene, the environment, modifier genes, and increased life expectancy [46]. In this context, in our data, the A allele is protective against osteoporosis, and is increased among young patients with unresolved *CFTR* mutation genotype. The osteoporosis frequency is shown in Table 6.

**Table 6:** **Age’s distribution and osteoporosis among *CFTR *mutation groups**

**Pancreatic status**	**Groups**	**N**	**Mean (months)**	**Standard deviation**	**Confidential interval**		**Minimum (months)**	**Maximum (months)**	**p-value**	**Osteoporosis (N/%)**	**p-value**
					**5%**	**95%**					
	No mutation identified	44	211.75	217.501	145.62	277.88	25	932		8 (19%)	
Pancreatic insufficiency + Pancreatic sufficiency	One *CFTR* mutation identified	51	201.18	165.050	154.76	247.60	11	782	0.854	9 (17.6%)	
	Two mutation identified	84	220.06	188.643	179.12	261.00	7	1274		12 (14.3%)	0.761
Pancreatic insufficiency	One *CFTR* mutation identified	35	221.57	216.17	147.31	295.83	25	932		7 (21.2%)	
	Two mutation identified	43	198.81	171.31	146.09	251.54	11	782	0.940	5 (11.6%)	0.345

*CFTR*, Cystic fibrosis transmembrane regulator; N, number of patients.

The role of the *GSTP1* polymorphism in CF hepatic disease has previously been analyzed [19]. The authors noted that CFTR protein expression was limited in liver epithelium; however, recent discoveries indicate that CFTR modulates the transport of GSH, creating a dysfunction in the antioxidant defense [47]. Of the liver detoxifying enzymes, GST plays a major role in protection against oxidative stress. The impact of *GSTM1* and *GSTP1* was also previously assessed in 106 CF patients where it was verified that the frequency of the GG genotype for the *GSTP1* + 313A > G polymorphism was significantly higher in CF patients with hepatic disease. This genotype was associated with an eight-fold increase in hepatic disease risk in patients younger than six years of age. These findings suggest that the identification of this polymorphism may have prognostic and awareness values for the treatment of CF patients with hepatic disease.

Considering the importance of the glutathione transport versus CFTR protein-mediated, patients with residual CFTR protein expression would have better performance in the extracellular oxidative stress response being favorable for the passage of GSH to the outside by residual CFTR activity. However, CF patients with two mutations screened in *CFTR* gene have principally alternate routes for the passing of GSH. Even taking into account that the most of GSH is transferred to the external environment via CFTR, in cases of residual CFTR (mutations Class IV, V and VI) would be modified slightly in relation to the GSH activity, since it is known that under 5% of CFTR expression occurs for minor severe *CFTR* mutations Classes, and approximately 65% of GSH passage occurs via CFTR, we had a percentage response to the GSH presence in external environment of at most 3.25% in cases of residual CFTR. Considering this factor, the analysis excluding the presence of PI patients enables better grouping of patients and optimizes the response of the associations found in our study.

Most studies analyzing the *GSTP1* gene related it to cancer and other diseases [22,30,44,48]. For example, the AA genotype of the *GSTP1* + 313A > G polymorphism was shown to offer protection against asthmatic symptoms [22]. Indeed, the *GSTP1* polymorphism was not previously found to affect pulmonary function in CF patients [30]. In an analysis of different genes involved in GST, there were no differences in GST activity and antioxidant levels observed between CF patients and controls. However, GST activity was lower in *P. aeruginosa*-infected CF children with severe clinical symptoms, as was the frequency of the *GSTP1* + 313A > G polymorphism AA genotype in uninfected (75%) compared with infected (33%) children [21]. It is possible that GST activity and *GSTP1* genotype play an important role in *P. aeruginosa* infection in CF patients. In support of this, the G allele of the *GSTP1* gene appears to be associated with an increased risk of severe pulmonary disease [21]. However, in a previous investigation into *GSTM1* and *GSTP1* polymorphisms in patients with CF and COPD, no significant associations were found between *GSTM1* activity and pulmonary disease severity. An analysis of genotypic combinations for *GSTM1* and *GSTP1* polymorphic loci showed that changes in *GSTP1* activities produced adverse effects in patients with COPD. Although *GSTM1* gene deletions may not themselves be implicated in pathogenesis, they may aggravate the disease in combination with *GSTP1* polymorphisms. Perhaps the strongest performance for the *GSTP1* gene in CF may result from the primary expression of this GST in the airways [48].

The present study showed that the homozygous deletion in *GSTT1* was a no mucoid *P. aeruginosa* risk factor in the no *CFTR* mutation group (*p* = 0.008; Table 2) and a protective factor for low values of SpO2. *GSTT1* expression is likely to act in the inflammatory response of the pulmonary parenchyma. As chronic airway infection by no mucoid *P. aeruginosa* is associated with greater clinical severity [49], the *GSTT1* polymorphism may be associated with the presence of *P. aeruginosa* through different mechanisms, including a low antioxidant response leading to further pulmonary degradation and the formation of a favorable environment for no mucoid *P. aeruginosa* colonization or infection.

The mechanism of gene action that determines which bacteria can colonize the lungs of CF patients is not fully understood. Similarly, it is also unclear which microorganisms are risk factors for the disease. Therefore, confirmation of a gene acting as modulator of an important metabolic pathway, such as GSH, may open up novel ways to identify the genetic factors that determine the severity of pulmonary disease. Future pharmacogenetic studies could then use this knowledge to provide new CF therapies.

Many previous studies have revealed that polymorphisms of *GSTM1* and *GSTT1* are associated with cancer [22,24,50,51], but few have been conducted in CF. Fifty-three children with CF were studied by Hull and Thomson [26], of which 26 with the *GSTM1* null allele had a significantly lower Shwachman-Kulczycki score. This supports the hypothesis that inflammation in CF contributes to tissue injury. Indeed, *GSTM1* null alleles can be a risk factor for pulmonary diseases in individuals with a reduced ability to deal with oxidants. There is also evidence that a high level of oxidative stress in the lungs of CF patients is caused by the release of reactive oxygen species by neutrophils [26]. In the present study, we found that expression of only one allele of *GSTM1* and *GSTT1* polymorphisms was associated with a low Bhalla score in patients with no *CFTR* mutation identified.

An interesting aspect was the high frequency of PS patients. The presence of PS occurred at exactly 20% of the sample. However, there was no difference distribution between the groups of patients with CF taking into account *CFTR* mutations groups (*p =* 0.621). Patients with two mutations identified in *CFTR* gene had 22.36% (19/85) of PS, values close to the other groups of patients [one identified mutation and no mutation identified with, respectively, 15.7% (8/51) and 20.5% (9/44)].

The PI is an important clinical marker of CF and is considered associated with the severity of disease and severe *CFTR* mutations (Class I, II and/or III). Studies considering populations of patients with CF, as performed by the Cystic Fibrosis Foundation give the prevalence of PI ranging from 5-10%. In our study, the high prevalence of PI may be associated with: (i) presence of higher frequency of mutations Class IV, V and/or VI, (ii) presence of modifier genes acting on the symptom of the disease, (iii) high miscegenation could be a protective factor for PI, (iv) environmental factor as an unknown protector.

The PI was used in statistical analysis as factor correction for no determination of *CFTR* mutation in CF groups with no or one *CFTR* mutation screened. After the patient exclusion to statistical analysis, all the previous positive associations were negative, except for *GSTM1* null allele. The null allele was associated as protector factor for onset of digestive symptoms (OR = 0.134; CI = 0.023-0.606; Table 2).

One important aspect considered was the age. Before the statistical analysis, the age was considered between the *CFTR* mutations groups (*p =* 0.854). The same occurred for *CFTR* mutations groups + insufficiency pancreatic (*p =* 0.940) (Table 6). No positive association was find considering age.

The divergent immune response is associated with multiple factors that denote the CF complexity such as the multigenic response, environmental influences, and interaction between airway microorganisms [49,52]. Clinically severe patients may have high initial inflammatory response, characterizing CF as a disease where inflammation occurs prior to infection [53]. Polymorphisms in genes that are involved in inflammation may be a risk factor for early severity of the disease [1], and patients with airways colonized by bacteria suffer early clinical deterioration and high levels of airway inflammation [54].

For the same population, a first study taking into account the same polymorphisms and clinical variables was performed. The previous data analyzed the genetic interaction among *GST* and *GCLC* polymorphisms, *CFTR* mutations and clinical markers. The data showed an interaction of *GSTM1* and *GSTT1* genes deletion, GSTP1* + 313A > G, and *CFTR* mutations (p = 0.008) and Bhalla clinical score by multifactor dimensionality reduction test. The Bhalla score is a computed tomography, which measures pulmonary involvement, therapeutic effects and selection of patients for transplantation, which detects anatomical changes of the lung parenchyma. The data published showed a first step to understand the complex mechanisms associated with the CF severity and modifier genes [55].

In the present study, we studied a CF population with complex clinical characteristics. By considering the different possible groupings of polymorphisms and clinical variables (Table 7) in relation to the *CFTR* gene, we performed various association studies. Supplementary data for *GCLC*-129C > T, *GCLC*-3506A > G, *GSTM1* gene deletion, *GSTT1* gene deletion, *GSTM1/GSTT1* gene deletions and GSTP1* + 313A > G are shown in Tables 8, 9, 10, 11, 12, 13 and 14. Further multicenter studies should be conducted to verify the influence of modifier genes in different *CFTR* genotypes.

**Table 7:** Patient characteristics (n = 180)

**Characteristic**	
Male gender	50% (90)
Age (months)	212 ± 15.75 (7–288)
Caucasoid	91.75%
BMI - thinness and accentuated thinness	22.22% (40)
One class I, II or III identified mutation	28.33% (51)
Two class I, II or III identified mutations	47.22% (85)
Age at first clinical manifestation (months)	35 ± 8.88 (0–156)
Age at diagnosis (months)	87 ± 13.63 (0–170.76)
Age at start of digestive symptoms (months)	40.6 ± 9.11 (0–149.4)
Age at start of pulmonary symptoms (months)	34.8 ± 9.88 (0–1156)
SpO2(%)	94.92 ± 4.26 (66–99)
Bhalla	8.74 ± 5.72 (0–25)
Kanga	18.85 ± 5.84 (10–40)
Shwachman-Kulczycki	65.85 ± 16.77 (20–95)
FVC(%)	79.29 ± 23.55 (19–135)
FEV_1_(%)	71.29 ± 27.467 (17–132)
FEV_1_/FVC(%)	83.46 ± 15.95 (37–137)
FEF_25–75_%	59.05 ± 35.55 (7–150)
Nasal polyps	18.33% (33)
Diabetes mellitus	18.33% (33)
Osteoporosis	16.11% (29)
Pancreatic insufficiency	80.0% (144)
Meconium ileus	15.00% (27)
Age at first isolated *P. aeruginosa* (months)	102.6 ± 14.45 (24–180)
*P. aeruginosa* status^a^	56.67% (102)
*P. aeruginosa* mucoid status^a^	42.22% (76)
*B. cepacia* status^a^	13.88% (25)
*A. xylosoxidans* status^a^	10.00% (18)
*S. aureus* status^a^	78.88% (142)

Continuous variables expressed as mean ± SD (range). Other data shown as percentage (number of patients). ^a^Based on three consecutive positive respiratory cultures.

N, Sample size; BMI, Body mass index; SpO2, Hemoglobin oxygen saturation in the blood; FVC, Forced vital capacity; FEV_1_, Forced expiratory volume in the first second; FEF_25–75_, Forced expiratory flow between 25 and 75% of FVC.

**Table 8:** **GCLC-129C > T polymorphism associated with CF clinical variables as distributed by *CFTR *mutation**

**Variable**	**Without taking *CFTR *** **mutation into account**		**No *CFTR *** **mutations identified**		**No *CFTR *** **mutations identified with PI**		**One identified *CFTR *** **mutation**		**One identified *CFTR *** **mutation with PI**		**Two identified *CFTR *** **mutations**	
	***p*-value**	***p*-corrected**	***p*-value**	***p*-corrected**	***p*-value**	***p*-corrected**	***p*-value**	***p*-corrected**	***p*-value**	***p*-corrected**	***p*-value**	***p*-corrected**
Gender^a^	0.577	1	1	1	1	1	0.024	0.096	0.698	1	0.418	1
Age^a^	0.348	1	1	1	0.667	1	1	1	1	1	0.249	0.996
Onset of symptoms^a^	1	1	0.165	0.660	0.401	1	1	1	1	1	0.392	1
Onset of pulmonary disease^a^	0.162	0.648	1	1	1	1	0.409	1	0.685	1	0.168	0.672
Onset of digestive disease^a^	1	1	0.142	0.568	0.354	1	0.710	1	0.650	1	0.583	1
Diagnosis^a^	1	1	1	1	0.405	1	0.715	1	0.412	1	0.764	1
BMI^a^	1	1	0.414	1	0.559	1	0.331	1	1	1	1	1
Bhalla score^b^	0.626	1	0.47	1	0.023	0.092	0.851	1	0.090	0.360	0.834	1
Kanga score^b^	0.277	1	0.45	1	0.632	1	0.687	1	0.625	1	0.192	0.768
Shwachman-Kulczycki score^b^	0.917	1	0.532	1	0.041	0.164	0.405	1	0.435	1	0.767	1
Nasal polyposis^a^	0.811	1	0.66	1	0.555	1	0.332	1	0.577	1	0.066	0.264
Diabetes mellitus^a^	0.811	1	1	1	1	1	1	1	0.315	1	0.505	1
Osteoporosis^a^	0.306	1	1	1	0.299	1	0.353	1	1	1	0.238	0.952
Meconium ileus	0.792	1	1	1	0.576	1	0.651	1	0.147	0.588	0.727	1
Pancreatic insufficiency^a^	0.063	0.252	0.267	1	-	-	0.328	1			1	1
SpO2^b^	0.384	1	0.296	1	0.078	0.312	0.124	0.496	0.864	1	0.597	1
FVC(%)^b^	0.822	1	0.828	1	0.127	0.508	0.922	1	0.506	1	0.597	1
FEV_1_(%)^b^	0.598	1	0.310	1	0.160	0.640	0.983	1	0.510	1	0.820	1
FEV_1_/FVC^b^	1	1	0.109	0.436	0.386	1	0.873	1	0.820	1	0.170	0.680
FEF_25–75_%^b^	0.448	1	0.044	0.176	1	1	0.982	1	0.767	1	0.537	1
First *P. aeruginosa*^a^	1	1	1	1	0.691	1	0.695	1	0.361	1	1	1
*P. aeruginosa* mucoid^a^	0.133	0.532	1	1	0.689	1	**0.011**	**0.044**	0.427	1	1	1
*P. aeruginosa* no mucoid^a^	1	1	1	1	1	1	0.266	1	0.680	1	0.391	1
*A. xylosoxidans*^a^	0.534	1	1	1	1	1	1	1	1	1	1	1
*S. aureus*^a^	0.261	1	0.093	0.372	1	1	1	1	1	1	1	1
*B. cepacia*^a^	1	1	1	1	1	1	1	1	0.318	1	1	1

^a^Fisher’s exact test used for categorical variables; ^b^Student’s t-test used for numerical variables. *P*-values < 0.05 denote clinical association (bold).

*CFTR*, Cystic fibrosis transmembrane regulator; PI, Pancreatic insufficiency; *GCLC*, Glutamate cysteine ligase catalytic subunit; BMI, Body mass index; SpO2, Hemoglobin oxygen saturation in the blood; FVC, Forced vital capacity; FEV_1_, Forced expiratory volume in the first second; FEF, Forced expiratory flow between 25 and 75% of vital capacity.

**Table 9:** **GCLC-3506A > G polymorphism in association with CF clinical variables as distributed by *CFTR *mutation**

**Variable**	**Without taking *CFTR *** **mutation into account**		**No *CFTR *** **mutations identified**		**No *CFTR *** **mutations identified with PI**		**One identified *CFTR *** **mutation**		**One identified *CFTR *** **mutation with PI**		**Two identified *CFTR *** **mutations**	
	***p*-value**	***p*-corrected**	***p*-value**	***p*-corrected**	***p*-value**	***p*-corrected**	***p*-value**	***p*-corrected**	***p*-value**	***p*-corrected**	***p*-value**	***p*-corrected**
Gender^a^	0.753	1	0.532	1	1	1	0.149	0.596	0.510	1	0.824	1
Age^a^	0.057	0.228	0.710	1	0.245	0.980	0.541	1	1	1	0.339	1
Onset of symptoms^a^	1	1	1	1	0.420	1	0.731	1	0.078	0.312	0.812	1
Onset of pulmonary disease^a^	0.507	1	1	1	0.152	0.608	0.727	1	0.302	1	0.816	1
Onset of digestive disease^a^	0.865	1	0.646	1	0.408	1	1	1	0.158	0.632	1	1
Diagnosis^a^	0.335	1	0.419	1	1	1	0.330	1	1	1	1	1
BMI^a^	1	1	1	1	0.281	1	0.704	1	0.709	1	0.785	1
Bhalla score^b^	0.35	1	0.830	1	0.468	1	0.169	0.676	0.833	1	0.495	1
Kanga score^b^	**0.011**	**0.044**	0.734	1	0.788		0.067	0.268	0.588	1	0.027	0.108
Shwachman-Kulczycki score^b^	0.091	0.364	0.725	1	0.223	0.892	0.034	0.136	0.545	1	0.159	0.636
Nasal polyposis^a^	0.688	1	0.251	1	0.555	1	0.692	1	1	1	0.083	0.332
Diabetes mellitus^a^	0.688	1	1	1	1	1	0.419	1	0.217	0.868	1	1
Osteoporosis^a^	0.133	0.532	1	1	0.068	0.272	0.25	1	0.630	1	0.335	
Meconium ileus	1	1	1	1	0.304	1	1	1	0.417	1	1	1
Pancreatic insufficiency^a^	0.698	1	0.180	0.720	-	-	0.376	1	-	-	1	1
SpO2^b^	0.033	0.132	0.234	0.936	0.142	0.568	0.548	1	0.134	0.536	0.149	0.596
FVC(%)^b^	0.412	1	0.944	1	0.061	0.244	0.036	0.144	0.755	1	0.955	1
FEV_1_(%)^b^	0.166	0.664	0.877	1	0.094	0.376	0.030	0.120	0.381	1	0.577	1
FEV_1_/FVC^b^	0.054	0.216	0.912	1	0.403	1	0.050	0.200	0.247	0.988	0.111	
FEF_25–75_%^b^	0.061	0.244	0.934	1	0.177	0.708	0.029	0.116	0.577	1	0.272	1
First *P. aeruginosa*^a^	0.350	1	0.453	1	0.433	1	0.716	1	0.015	0.060	0.799	1
*P. aeruginosa* mucoid^a^	0.152	0.608	1	1	0.443	1	0.064	0.256	0.178	0.712	0.371	1
*P. aeruginosa* no mucoid^a^	0.057	0.228	1	1	0.243	0.972	**0.003**	**0.012**	0.023	0.092	0.351	1
*A. xylosoxidans*^a^	0.187	0.748	0.066		0.044	0.176	0.565	1	1	1	0.343	1
*S. aureus*^a^	0.849	1	0.708	1	1	1	0.376	1	0.024	0.096	0.394	1
*B. cepacia*^a^	0.246	0.984	1	1	1	1	0.471	1	0.082	0.328	0.394	1

^a^Fisher’s exact test used for categorical variables; ^b^Student’s t-test used for numerical variables. *P*-values < 0.05 denote clinical association (bold).

*CFTR*, Cystic fibrosis transmembrane regulator; *GCLC*, Glutamate cysteine ligase catalytic subunit; PI, Pancreatic insufficiency; BMI, Body mass index; SpO2, Hemoglobin oxygen saturation in the blood; FVC, Forced vital capacity; FEV_1_, Forced expiratory volume in the first second; FEF, Forced expiratory flow between 25 and 75% of vital capacity.

**Table 10:** **GCLC-129C > T and GCLC-3506A > G polymorphisms by haplotype in association with CF clinical variables as distributed by *CFTR *mutation**

**Variable**	**Without taking *CFTR *** **mutation into account**		**No *CFTR *** **mutations identified**		**No *CFTR *** **mutations identified with PI**		**One identified *CFTR *** **mutation**		**One identified *CFTR *** **mutation with PI**		**Two identified *CFTR *** **mutations**	
	***p*-value**	***p*-corrected**	***p*-value**	***p*-corrected**	***p*-value**	***p*-corrected**	***p*-value**	***p*-corrected**	***p*-value**	***p*-corrected**	***p*-value**	***p*-corrected**
Gender^a^	0.285	1	0.302	1	0.505	1	0.424	1	0.479	1	0.223	0.892
Age^a^	0.437	1	0.639	1	0.382	1	0.601	1	0.777	1	0.625	1
Onset of symptoms^a^	0.098	0.392	0.407	1	0.421	1	0.143	0.572	0.180	0.720	0.036	0.144
Onset of pulmonary disease^a^	0.802	1	0.091	0.364	0.163	0.652	0.424	1	0.321	1	0.980	1
Onset of digestive disease^a^	0.640	1	0.237	0.948	0.311	1	0.692	1	0.318	1	0.452	1
Diagnosis^a^	0.334	1	0.620	1	0.637	1	0.715	1	0.613	1	0.218	0.872
BMI^a^	0.620	1	0.376	1	0.510	1	0.848	1	0.754	1	0.665	1
Bhalla score^b^	0.942	1	0.808	1	0.158	0.632	0.830	1	0.324	1	0.311	1
Kanga score^b^	0.879	1	0.884	1	0.805	1	0.753	1	0.745	1	0.822	1
Shwachman-Kulczycki score^b^	0.985	1	0.416	1	0.151	0.604	0.538	1	0.837	1	0.981	1
Nasal polyposis^a^	0.582	1	0.347	1	0.285	1	0.457	1	0.631	1	0.986	1
Diabetes mellitus^a^	0.255	1	0.858	1	0.917	1	0.201	0.804	0.337	1	0.383	1
Osteoporosis^a^	0.562	1	0.829	1	0.108	0.432	0.339	1	0.036	0.144	**0.713**	**1**
Meconium ileus	0.420	1	0.389	1	0.379	1	0.130	0.520	0.080	0.320	0.619	1
Pancreatic insufficiency^a^	0.159	0.636	0.398	1	-	-	0.167	0.668	-	-	0.601	1
SpO2^b^	0.506	1	0.201	0.804	0.128	0.512	0.422	1	0.525	1	0.278	1
FVC(%)^b^	0.498	1	0.216	0.864	0.121	0.484	0.738	1	0.901	1	0.499	1
FEV_1_(%)^b^	0.668	1	0.214	0.856	0.201	0.804	0.479	1	0.731	1	0.769	1
FEV_1_/FVC^b^	0.615	1	0.592	1	0.671	1	0.407	1	0.686	1	0.373	1
FEF_25–75_%^b^	0.643	1	0.326	1	0.531	1	0.548	1	0.942	1	0.851	1
First *P. aeruginosa*^*a*^	0.147	0.588	0.125	0.500	0.341	1	0.146	0.584	0.027	0.108	0.264	1
*P. aeruginosa* mucoid^a^	0.559	1	0.316	1	0.366	1	0.569	1	0.285	1	0.160	0.640
*P. aeruginosa* no mucoid^a^	0.319	1	0.263	1	0.352	1	0.276	1	0.082	0.328	0.347	1
*A. xylosoxidans*^*a*^	0.327	1	0.018	0.072	0.013	0.052	0.687	1	0.646	1	**0.006**	**0.024**
*S. aureus*^*a*^	0.843	1	0.677	1	0.931	1	0.049	0.196	0.032	0.128	0.466	1
*B. cepacia*^*a*^	0.461	1	0.734	1	0.974	1	0.150	0.600	0.243	0.972	0.671	1

^a^Fisher’s exact test used for categorical variables; ^b^Student’s t-test used for numerical variables. *P*-values < 0.05 denote clinical association (bold).

*CFTR*, Cystic fibrosis transmembrane regulator; *GCLC*, Glutamate cysteine ligase catalytic subunit; PI, Pancreatic insufficiency; BMI, Body mass index; SpO2, Hemoglobin oxygen saturation in the blood; FVC, Forced vital capacity; FEV_1_, Forced expiratory volume in the first second; FEF, Forced expiratory flow between 25 and 75% of vital capacity.

**Table 11:** ***GSTM1 *deletion polymorphism in association with CF clinical variables as distributed by *CFTR *mutation**

**Variable**	**Without taking *CFTR *** **mutation into account**		**No *CFTR *** **mutations identified**		**No *CFTR *** **mutations identified with PI**		**One identified *CFTR *** **mutation**		**One identified *CFTR *** **mutation with PI**		**Two identified *CFTR *** **mutations**	
	***p*-value**	***p*-corrected**	***p*-value**	***p*-corrected**	***p*-value**	***p*-corrected**	***p*-value**	***p*-corrected**	***p*-value**	***p*-corrected**	***p*-value**	***p*-corrected**
Gender^a^	0.171	0.684	0.764	1	0.075	0.300	0.267	1	0.537	1	0.110	0.440
Age^a^	1	1	0.498	1	0.736	1	0.579	1	1	1	1	1
Onset of symptoms^a^	0.268	1	0.459	1	0.721	1	1	1	1	1	0.629	1
Onset of pulmonary disease^a^	0.424	1	0.068	0.272	1	1	1	1	0.742	1	0.639	1
Onset of digestive disease^a^	0.409	1	0.665	1	1	1	1	1	**0.008**	**0.032**	0.635	1
Diagnosis^a^	1	1	0.059	0.236	0.729	1	0.149	0.596	1	1	1	
BMI^a^	0.462	1	0.503	1	0.115	0.460	0.725	1	1	1	0.169	0.676
Bhalla score^b^	0.86	1	0.11	0.44	0.059	0.236	0.050	0.200	0.692	1	0.879	1
Kanga score^b^	0.982	1	0.693	1	0.367	1	0.822	1	0.480	1	0.784	1
Shwachman-Kulczycki score^b^	0.501	1	0.449	1	0.884	1	0.123	0.492	0.777	1	0.568	1
Nasal polyposis^a^	0.331	1	0.136	0.544	0.610	1	1	1	0.407	1	0.765	1
Diabetes mellitus^a^	0.560	1	1	1	1	1	0.703	1	1	1	0.169	0.676
Osteoporosis^a^	0.217	0.868	0.435	1	0.377	1	0.173		0.633	1	1	1
Meconium ileus	1	1	1	1	0.640	1	0.726	1	1	1	0.776	1
Pancreatic insufficiency^a^	1	1	0.765	1	-	-	1	1	-	-	1	1
SpO2^b^	0.187	0.748	**0.012**	**0.048**	0.500	1	0.780	1	0.652	1	0.645	1
FVC(%)^b^	0.990	1	0.741	1	0.020	0.08	0.538	1	0.307	1	0.967	1
FEV_1_(%)^b^	0.827	1	0.623	1	0.030	0.012	0.786	1	0.972	1	0.943	1
FEV_1_/FVC^b^	0.915	1	0.749	1	0.532	1	0.918	1	0.244	0.976	0.597	1
FEF_25–75_%^b^	0.853	1	0.718	1	0.197	0.788	0.819	1	0.255	1	0.847	1
First *P. aeruginosa*^a^	0.724	1	1	1	0.473	1	0.056	0.224	1	1	0.312	1
*P. aeruginosa* mucoid^a^	0.092	0.368	0.729	1	0.289	1	1	1	0.541	1	0.107	0.428
*P. aeruginosa* no mucoid^a^	0.879	1	0.754	1	0292	1	0.776	1	1	1	0.629	1
*A. xylosoxidans*^*a*^	0.619	1	0.537	1	1	1	0.35	1	0.511	1	0.52	1
*S. aureus*^*a*^	0.362	1	0.175	0.700	0.391	1	1	1	1	1	0.776	1
*B. cepacia*^*a*^	0.371	1	0.116	0.464	0.313	1	0.703	1	1	1	1	1

^a^Fisher’s exact test used for categorical variables; ^b^Student’s t-test used for numerical variables. *P*-values < 0.05 denote clinical association (bold).

*CFTR*, Cystic fibrosis transmembrane regulator; *GSTM1*, Glutathione S-transferase mu 1; PI, Pancreatic insufficiency; BMI, Body mass index; SpO2, Hemoglobin oxygen saturation in the blood; FVC, Forced vital capacity; FEV_1_, Forced expiratory volume in the first second; FEF, Forced expiratory flow between 25 and 75% of vital capacity.

**Table 12:** ***GSTT1 *deletion polymorphism in association with CF clinical variables as distributed by *CFTR *mutation**

**Variable**	**Without taking *CFTR *mutation into account**		**No *CFTR *mutations identified**		**No *CFTR *mutations identified with PI**		**One identified *CFTR *mutation**		**One identified *CFTR *mutation with PI**		**Two identified *CFTR *mutations**	
	***p*-value**	***p*-corrected**	***p*-value**	***p*-corrected**	***p*-value**	***p*-corrected**	***p*-value**	***p*-corrected**	***p*-value**	***p*-corrected**	***p*-value**	***p*-corrected**
Gender^a^	0.211	0.844	0.778	1	0.378	1	1	1	0.215	0.860	0.081	0.324
Age^a^	0.043	0.166	0.750	1	0.505	1	0.083	0.332	0.531	1	0.795	0.795
Onset of symptoms^a^	1	1	0.305	1	1	1	0.202	0.808	0.746	1	0.600	1
Onset of pulmonary disease^a^	0.620	1	0.721	1	0.229	0.916	0.521	1	0.746	1	0.796	1
Onset of digestive disease^a^	0.863	1	0.390	1	1	1	0.344	1	0.179	0.716	1	1
Diagnosis^a^	0.745	1	1	1	0.303	1	0.068	0.272	0.537	1	0.779	1
BMI^a^	0.447	1	0.747	1	0.103	0.412	0.295	1	0.728	1	0.537	1
Bhalla score^b^	0.485	1	0.824	1	0.134	0.536	0.322	1	0.634	1	0.185	0.740
Kanga score^b^	0.737	1	0.743	1	0.421	1	0.953	1	0.321	1	0.767	1
Shwachman-Kulczycki score^b^	0.734	1	0.984	1	0.013	0.052	0.653	1	0.925	1	0.393	1
Nasal polyposis^a^	0.313	1	1	1	1	1	0.062	0.248	0.685	1	1	1
Diabetes mellitus^a^	0.158	0.632	0.115	0.460	0.398	1	0.450	1	0.071	0.284	0.764	1
Osteoporosis^a^	1	1	1	1	0.085	0.340	1	1	0.230	0.920	0.718	1
Meconium ileus	0.276	1	0.077	0.308	0.658	1	1	1	0.445	1	0.335	1
Pancreatic insufficiency^a^	0.847	1	0.561	1	-	-	1	1	-	-	0.557	1
SaO2^b^	0.988	1	0.740	1	0.170	0.680	0.595	1	0.333	1	0.703	1
FVC(%)^b^	0.268	1	0.086	0.344	0.154	0.616	0.464	1	0.412	1	0.623	1
FEV_1_(%)^b^	0.310	1	0.167	0.668	0.029	0.116	0.564	1	0.597	1	0.636	1
FEV_1_/FVC^b^	0.404	1	0.288	1	0.017	0.068	0.692	1	0.676	1	0.424	1
FEF_25–75_%^b^	0.687	1	0.390	1	0.027	0.108	0.686	1	0.829	1	0.959	1
First *P. aeruginosa*^*a*^	0.472	1	1	1	0.724	1	0.320	1	0.713	1	0.085	0.340
*P. aeruginosa* mucoid^a^	0.433	1	0.747	1	1	1	0.393	1	0.753	1	1	1
*P. aeruginosa* no mucoid^a^	0.876	1	**0.002**	**0.008**	0.489	1	0.133	0.266	0.541	1	1	1
*A. xylosoxidans*^*a*^	0.437	1	1	1	0.338	1	0.623	1	0.151	0.604	0.295	1
*S. aureus*^*a*^	0.705	1	1	1	0.658	1	0.325	1	0.735	1	1	1
*B. cepacia*^*a*^	1	1	1	1	0.177	0.708	0.699	1	0.407	1	0.215	0.860

^a^Fisher’s exact test used for categorical variables; ^b^Student’s t-test used for numerical variables. *P*-values < 0.05 denote clinical association (bold).

*CFTR*, Cystic fibrosis transmembrane regulator; *GSTT1*, Glutathione S-transferase theta 1; PI, Pancreatic insufficiency; BMI, Body mass index; SpO2, Hemoglobin oxygen saturation in the blood; FVC, Forced vital capacity; FEV_1_, Forced expiratory volume in the first second; FEF, Forced expiratory flow between 25 and 75% of vital capacity.

**Table 13:** ***GSTM1/GSTT1 *deletion polymorphism in association with CF clinical variables as distributed by *CFTR *mutation**

**Variable**	**Without taking *CFTR *** **mutation into account**		**No *CFTR *** **mutations identified**		**No *CFTR *** **mutations identified with PI**		**One identified *CFTR *** **mutation**		**One identified *CFTR *** **mutation with PI**		**Two identified *CFTR *** **mutations**	
	***p*-value**	***p*-corrected**	***p*-value**	***p*-corrected**	***p*-value**	***p*-corrected**	***p*-value**	***p*-corrected**	***p*-value**	***p*-corrected**	***p*-value**	***p*-corrected**
Gender^a^	0.036	0.144	0.943	1	0.601	1	0.369	1	0.114	0.456	0.014	0.056
Age^a^	0.331	1	0.647	1	0.496	1	0.054	0.216	0.149	0.596	0.908	1
Onset of symptoms^a^	0.300	1	0.996	1	0.835	1	0.579	1	0.854	1	0.049	0.196
Onset of pulmonary disease^a^	0.588	1	0.359	1	0.431	1	0.776	1	0.267	1	0.559	1
Onset of digestive disease^a^	0.626	1	0.480	1	0.581	1	0.433	1	0.458	1	0.051	0.204
Diagnosis^a^	0.520	1	0.207	0.828	0.490	1	0.710	1	0.510	1	0.992	1
BMI^a^	0.283	1	0.954	1	0.717	1	0.252	1	0.998	1	0.596	1
Bhalla score^b^	0.088	0.352	**0.005**	**0.02**	0.915	1	0.381	1	0.218	0.872	0.481	1
Kanga score^b^	0.885	1	0.443	1	0.216	0.864	0.912	1	0.261	1	0.455	1
Shwachman-Kulczycki score^b^	0.627	1	0.144	0.576	0.087	0.348	0.387	1	0.104	0.416	0.195	0.780
Nasal polyposis^a^	0.098	0.392	0.483	1	0.699	1	0.467	1	0.362	1	0.102	0.408
Diabetes mellitus^a^	0.259	1	0.240	0.96	0.790	1	0.992	1	0.555	1	0.334	1
Osteoporosis^a^	0.204	0.816	0.501	1	0.525	1	0.427	1	0.187	0.748	0.386	1
Meconium ileus	0.683	1	0.266	1	0.348	1	0.517	1	0.905	1	0.626	1
Pancreatic insufficiency^a^	0.965	1	0.791	1	-	-	0.975	1	-	-	0.653	1
SpO2^b^	0.449	1	0.021	0.084	0.557	1	0.616	1	0.774	1	0.786	1
FVC(%)^b^	0.576	1	0.518	1	0.859	1	0.928	1	0.475	1	0.758	1
FEV_1_(%)^b^	0.778	1	0.182	0.728	0.977	1	0.799	1	0.827	1	0.657	1
FEV_1_/FVC^b^	0.178	1	0.007	0.028	0.265	1	0.789	1	0.395	1	0.593	1
FEF_25–75_%^b^	0.881	1	0.014	0.056	0.751	1	0.719	1	0.370	1	0.382	1
First *P. aeruginosa*^*a*^	0.545	1	0.686	1	0.295	1	0.019	0.076	0.299	1	0.123	0.492
*P. aeruginosa* mucoid^a^	0.134	0.536	0.118	0.472	0.492	1	0.575	1	0.940	1	0.337	1
*P. aeruginosa* no mucoid^a^	0.487	1	0.051	0.204	0.682	1	0.167	0.668	0.190	0.760	0.847	1
*A. xylosoxidans*^*a*^	0.541	1	0.779	1	0.663	1	0.079	1	0.537	1	0.995	1
*S. aureus*^*a*^	0.660	1	0.243	0.972	0.839	1	0.716	1	0.181	0.724	0.667	1
*B. cepacia*^*a*^	0.861	1	0.142	0.568	0.054	1	0.759	1	0.145	0.580	0.640	1

^a^Fisher’s exact test used for categorical variables; ^b^Student’s t-test used for numerical variables. *P*-values < 0.05 denote clinical association (bold).

*CFTR*, Cystic fibrosis transmembrane regulator; *GSTM1*, Glutathione S-transferase mu 1; *GSTT1*, Glutathione S-transferase theta 1; PI, Pancreatic insufficiency; BMI, Body mass index; SpO2, Hemoglobin oxygen saturation in the blood; FVC, Forced vital capacity; FEV_1_, Forced expiratory volume in the first second; FEF, Forced expiratory flow between 25 and 75% of vital capacity.

**Table 14:** **GSTP1 + 313A > G polymorphism in association with CF clinical variables as distributed by *CFTR *mutation**

**Variable**	**Without taking *CFTR *** **mutation into account**		**No *CFTR *** **mutations identified**		**No *CFTR *** **mutations identified with PI**		**One identified *CFTR *** **mutation**		**One identified *CFTR *** **mutation with PI**		**Two identified *CFTR *** **mutations**	
	***p*-value**	***p*-corrected**	***p*-value**	***p*-corrected**	***p*-value**	***p*-corrected**	***p*-value**	***p*-corrected**	***p*-value**	***p*-corrected**	***p*-value**	***p*-corrected**
Gender^a^	0.550	1	0.396	1	1	1	0.267	1	0.763	1	0.184	0.736
Age^a^	**0.011**	**0.044**	0.750	1	0.500	1	0.051	0.204	1	1	0.058	0.232
Onset of symptoms^a^	0.876	1	0.473	1	0.720	1	1	1	0.531	1	1	1
Onset of pulmonary disease^a^	0.754	1	0.729	1	0.252	1	0.757	1	0.757	1	1	1
Onset of digestive disease^a^	0.516	1	1	1	0.462	1	0.761	1	1	1	1	1
Diagnosis^a^	0.644	1	0.694	1	0.185	0.740	0.561	1	0.763	1	0.441	1
BMI^a^	0.856	1	0.331	1	1	1	1	1	0.488	1	1	1
Bhalla score^b^	0.098	0.392	0.187	1	0.671	1	0.491	1	0.098	0.392	0.392	1
Kanga score^b^	0.716	1	0.867	1	0.604	1	0.407	1	0.416	1	0.300	1
Shwachman-Kulczycki score^b^	0.554	1	0.984	1	0.121	0.484	0.73	1	0.198	0.792	0.170	0.680
Nasal polyposis^a^	0.848	1	0.306	1	0.601	1	1	1	0.412	1	0.562	1
Diabetes mellitus^a^	0.336	1	1	1	1	1	0.703	1	1	1	0.582	1
Osteoporosis^a^	0.159	0.318	0.715	0.953			1	1	0.345	1	**0.009**	**0.036**
Meconium ileus	0.403	1	1	1	0.398	1	1	1	0.457	1	0.161	0.644
Pancreatic insufficiency^a^	0.581	1	0.393	1	0.187	0.748	0.703	1	-	-	0.578	1
SpO2^b^	0.967	1	0.839	1	0.230	0.920	0.156	0.624	0.157	0.628	0.346	1
FVC(%)^b^	0.441	1	0.407	1	0.279	1	0.849	1	0.315	1	0.626	1
FEV_1_(%)^b^	0.338	1	0.467	1	0.923	1	0.907	1	0.221	0.884	0.451	1
FEV_1_/FVC^b^	0.295	1	0.265	1	0.218	0.872	0.575	1	0.771	1	0.439	1
FEF_25–75_%^b^	0.146	0.584	0.498	1	0.261	1	0.505	1	0.379	1	0.291	1
First *P. aeruginosa*^*a*^	0.035	0.140	1	1	0.473	1	0.056	0.224	1	1	0.203	0.812
*P. aeruginosa* mucoid^a^	0.289	1	0.331	1	0.505	1	0.782	1	0.760	1	0.653	1
*P. aeruginosa* no mucoid^a^	1	1	0.548	1	1	1	0.776	1	0.760	1	0.482	1
*A. xylosoxidans*^*a*^	0.806	1	0.196	0.784	1	1	0.350	1	0.488	1	0.755	1
*S. aureus*^*a*^	0.721	1	0.507	1	1	1	0.743	1	0.185	0.740	0.565	1
*B. cepacia*^*a*^	0.667	1	0.196	0.784	1	1	0.703	1	0.698	1	0.404	1

^a^Fisher’s exact test used for categorical variables; ^b^Student’s t-test used for numerical variables. *P*-values < 0.05 denote clinical association (bold).

*CFTR*, Cystic fibrosis transmembrane regulator; *GSTP1*, Glutathione S-transferase pi 1; PI, Pancreatic insufficiency; BMI, Body mass index; SpO2, Hemoglobin oxygen saturation in the blood; FVC, Forced vital capacity; FEV_1_, Forced expiratory volume in the first second; FEF, Forced expiratory flow between 25 and 75% of vital capacity.

Study limitations: (i) *CFTR* mutation with no complete screening; (ii) short population of CF patients; (iii) spirometry test performed by transversal method and did no performed longitudinally; (iv) no measure of GSH activity or GST and GCLC proteins, taking into account the sample collection limitation in our center and time to process all data. Study highlights the data by: (i) one CF center collection – considering an admixed population, the CF patients from one center minimizes miscegenation factors. Another fact, is the similar environmental and the same access to treatment; (ii) high number of clinical markers evaluated provides better association and characterization of modifier genes action; (iii) complete CF diagnosis performed by different methods.

## Conclusions

Our results show that, although a monogenic disease, CF is heavily influenced in its clinical characteristics, evolution and severity by polymorphisms in modifier genes. Nevertheless, there is still a long way before the dynamics of polymorphisms in genes active in the GSH metabolic pathway and involved in detoxification in CF are fully understood.

Another fact is the prevalence of PS and PI that should be considered in all studies in the future, being associated with different phenotype and genotype.

## Abbreviations

CF: Cystic fibrosis; CFTR: Cystic fibrosis transmembrane regulator; GCLC: Glutamate-cysteine ligase, catalytic subunit; GST: Glutathione S-transferase; GSTM1: Glutathione S-transferase mu 1; GSTT1: Glutathione S-transferase tetha 1; GSTP1: Glutathione S-transferase pi 1; NMPA: No mucoid *Pseudomonas aeruginosa*; SpO2: Transcutaneous hemoglobin oxygen saturation; FEV1%: Forced expiratory volume in 1 second; FVC: Forced vital capacity; FEF25-75%: Forced expiratory flow 25–75%; BMI: Body mass index; WHO: World health organization; MLPA: Multiplex ligation-dependent probe amplification; PCR: Polymerase chain reaction; SPSS: Statistical package for social science for windows; PS: Pancreatic sufficiency; PI: Pancreatic insufficiency; COPD: Chronic obstructive pulmonary disease.

## Competing interests

The authors declare that they have no competing interests.

## Authors’ contributions

FALM contributed to the study conception and design, acquired, analyzed and interpreted the data, drafted the manuscript and revised it for intellectual content. CSB carried out the molecular genetic studies and drafted the manuscript. AFR drafted the manuscript and revised it for intellectual content. JDR approved the manuscript for publication. All authors read and approved the final manuscript.

## Pre-publication history

The pre-publication history for this paper can be accessed here:

http://www.biomedcentral.com/1471-2350/15/27/prepub
